# Global trends and gaps in pharmacogenetic clinical trials: A cross-sectional analysis of ClinicalTrials.gov registrations

**DOI:** 10.1097/MD.0000000000046574

**Published:** 2025-12-19

**Authors:** Mohammed Aldurdunji, Ahmed Ashour, Fahad Alshehri, Nasser Alorfi

**Affiliations:** a Pharmaceutical Practices Department, College of Pharmacy, Umm Al-Qura University, Makkah, Saudi Arabia; b Pharmacology and Toxicology Department, College of Pharmacy, Umm Al-Qura University, Makkah, Saudi Arabia.

**Keywords:** clinical trials, ClinicalTrials.gov, personalized therapy, pharmacogenetics, precision medicine

## Abstract

Pharmacogenetics has emerged role in precision medicine, yet clinical translation remains limited. Understanding the scope and focus of clinical trials is crucial to inform future implementation efforts. This study aims to evaluate the characteristics, trends, and thematic gaps of pharmacogenetic clinical trials registered on ClinicalTrials.gov. A cross-sectional descriptive analysis was conducted on interventional pharmacogenetic trials registered through 2025. Trials were identified using targeted keyword searches, and data were extracted on study phase, condition, intervention, population, and recruitment status. Frequencies and percentages were computed to describe trends and distributions. A total of 1743 pharmacogenetic and pharmacogenomic clinical trials were included. Oncology accounted for the largest share (30.1%), followed by pharmacogenetics/general (19.4%) and infectious diseases (10.3%). Phase 1 studies were the most common (45.0%), followed by Phase 2 (26.2%) and Phase 4 (20.7%). The majority of trials were reported as completed (68.9%), while smaller proportions were classified as recruiting (5.8%) or terminated (6.6%). Trial registrations peaked between 2008 and 2015 and declined thereafter. Oncology drugs, particularly irinotecan, were the most frequently studied agents, followed by antimicrobials and cardiovascular drugs. Pharmacogenetic research has expanded significantly since the early 2000s, but imbalances remain across disease areas. These Gaps highlight the need for more inclusive and diverse study designs. Addressing educational, infrastructural, and regulatory barriers is critical to advance clinical adoption.

## 1. Introduction

Pharmacogenetics has been established as a fundamental component of precision medicine, with efforts directed towards elucidating the genetic basis of interindividual variability in drug response. Increasing emphasis has been placed on the translation of pharmacogenetic findings into clinical practice, aiming to enhance therapeutic outcomes and reduce adverse drug reactions. The validation of pharmacogenetic associations and demonstration of their clinical utility and cost-effectiveness across diverse populations and therapeutic contexts have been pursued primarily through clinical trials.^[1]^

The systematic evaluation of pharmacogenetic interventions has been recognized as essential for establishing evidence-based personalized therapies. Randomized controlled trials evaluating genotype-guided dosing algorithms, such as those involving CYP2C9 and vitamin K epoxide reductase complex subunit 1 variants for warfarin, have provided evidence for improved dosing accuracy and reduced complications.^[2,3]^ Furthermore, the integration of pharmacogenetic profiles into clinical decision support systems has been shown to improve medication management in populations at elevated risk for adverse drug reactions.^[4]^

Considerable heterogeneity exists in the design, quality, and reporting standards of pharmacogenetic clinical trials worldwide. Many studies have concentrated on narrowly defined drugs, genetic markers, or populations, producing a fragmented and sometimes non-generalizable evidence base.^[5,6]^ This variability underscores the importance of comprehensive synthesis and the development of rigorous reporting frameworks, such as the Strengthening the Reporting of Pharmacogenetic Studies guideline, to improve consistency and transparency in trial reporting.^[7]^ ClinicalTrials.gov has emerged as a cornerstone resource for systematically identifying and evaluating pharmacogenetic and pharmacogenomic research, enabling broad-based analyses that can illuminate emerging trends and persistent gaps in the field.^[8]^ Recent systematic evaluations have further emphasized the need for harmonized methodologies to ensure that pharmacogenetic interventions are appropriately validated and clinically translatable.^[6,9]^

Barriers to clinical implementation of pharmacogenetics have been identified, including insufficient provider education and awareness, which limit the uptake of pharmacogenetic testing.^[10]^ Additionally, challenges related to integrating genetic data into electronic health record systems have impeded clinical decision-making processes.^[11]^ Economic factors, particularly inconsistent insurance reimbursement, have also restricted patient access to testing despite demonstrated clinical benefits.^[12]^ Ethical and regulatory considerations further complicate implementation, necessitating the development of standardized guidelines to ensure safe and effective use.^[13,14]^ Positive attitudes towards pharmacogenomics among healthcare professionals, including pediatricians, have been documented, although practical implementation remains limited.^[12]^

To address these challenges, the expansion of pharmacogenetic research to include underrepresented therapeutic areas and, particularly, diverse ethnic groups, has been recommended.^[15]^ The incorporation of diverse real-world data and pragmatic clinical trial designs has been proposed to enhance recruitment and outcome assessment.^[16]^ International collaboration and stakeholder engagement have been suggested as means to facilitate the translation of pharmacogenetic findings into clinical practice.^[17]^ Moreover, advances in artificial intelligence and machine learning have been recognized as promising tools for optimizing data analysis and clinical implementation strategies.^[18,19]^

The present study aims to systematically identify, categorize, and analyze pharmacogenetic clinical trials registered on ClinicalTrials.gov. Trial characteristics, methodological trends, disease categories, and drug classes are described to highlight the current landscape of pharmacogenetic research and identify opportunities for clinical integration.

## 2. Methods

### 2.1. Study design

This study was conducted as a cross-sectional, descriptive analysis of pharmacogenetic clinical trials registered on ClinicalTrials.gov. The analysis was designed to characterize the landscape and trends in pharmacogenetic research by systematically identifying eligible trials and extracting relevant study characteristics.

### 2.2. Data source and search strategy

ClinicalTrials.gov served as the primary data source for identifying relevant clinical trials. The advanced search function was used with the terms pharmacogenetic* OR pharmacogenomic* OR “genotype-guided” OR “genotype guided” OR CYP2D6 OR CYP2C19 OR CYP2C9 OR CYP2B6 OR CYP3A5 OR thiopurine S-methyltransferase OR NUDT15 OR UDP-glucuronosyltransferase 1A1 (UGT1A1) OR DPYD OR vitamin K epoxide reductase complex subunit 1 OR SLCO1B1 OR HLA-B57:01 OR HLA-B15:02 OR HLA-A*31:01 OR G6PD in any part of the study record.

The search was restricted to interventional studies with designated phases (Early Phase 1, Phase 1, Phase 2, Phase 3, and Phase 4). No restrictions were placed on recruitment status, location, or sponsor type. The search was performed on September 06, 2025, and yielded a total of 203 studies meeting the initial inclusion criteria.

### 2.3. Eligibility criteria

Studies were included if they^[1]^ were registered on ClinicalTrials.gov,^[2]^ described pharmacogenetic or pharmacogenomic interventions or analyses,^[3]^ involved human participants, and were assigned to a designated clinical trial phase (Early Phase 1–4). Trials focused exclusively on other types of genetic research without a pharmacogenetic component were excluded. Both completed and ongoing trials were eligible.

### 2.4. Data extraction

The following data were extracted from each eligible study: ClinicalTrials.gov identifier, study title, recruitment status, trial phase, study design, condition or disease area, sample size, geographic location, funding source, start and completion dates, genes or genetic variants investigated, intervention or drug studied, and primary outcome measures. Data extraction was performed using a standardized form.

### 2.5. Data analysis

Descriptive statistics were used to summarize the general characteristics of the included trials. Frequencies and percentages were calculated for categorical variables such as study phase, recruitment status, disease category, and drug class. Continuous variables such as sample size were summarized using medians and ranges. Temporal trends in pharmacogenetic trial registration and distributions by disease category, intervention, and drug class were assessed. All analyses were performed using Microsoft Excel (version 365). Results are presented in tabular and graphical formats where appropriate.

## 3. Results

### 3.1. Overview of included studies and disease categories

The distribution of condition–study pairs across the 1743 included pharmacogenetic and pharmacogenomic clinical trials is summarized in Table 1. Three categories accounted for the majority of studies: oncology (524 entries, 30.0%), pharmacogenetics/general (339 entries, 19.4%), and infectious diseases (180 entries, 10.3%). Within these domains, the most frequently studied subconditions were cancer (general), colorectal cancer, and breast cancer in oncology; healthy volunteers in pharmacogenetics/general; and malaria, HIV infection, and tuberculosis in infectious diseases.

**Table 1:** Distribution of pharmacogenetic and pharmacogenomic clinical trials by disease category and leading subconditions. Counts represent the number of study–condition pairs identified among 1746 condition entries from 1743 pharmacogenetic clinical trials registered on ClinicalTrials.gov. For each main category, the 3 most frequent subconditions are shown.

Main category	Subcondition	Count
Oncology (n = 524)	Cancer (general)	42
	Colorectal cancer	36
	Breast cancer	35
Pharmacogenetics/general (n = 339)	Healthy volunteers	253
	Pharmacogenetics/general	22
	Pharmacokinetics	13
Infectious diseases (n = 183)	Malaria	60
	HIV infection	43
	Tuberculosis	13
Psychiatric/neurological (n = 112)	Schizophrenia	25
	Major depressive disorder	24
	Depression	7
Cardiovascular (n = 106)	Coronary artery disease	48
	Atrial fibrillation	13
	Hypertension	11
Metabolic/endocrine (n = 71)	Type 2 diabetes mellitus	16
	Obesity	10
	Crohn disease	10
Musculoskeletal (n = 44)	Pain	16
	Rheumatoid arthritis	7
	Chronic pain	4
Transplantation/immunology (n = 35)	Kidney transplantation	15
	Transplant complications	7
	Kidney disease	3
Respiratory (n = 32)	Asthma	14
	Chronic obstructive pulmonary disease	8
	Dyspnea	2
Substance use (n = 30)	Alcoholism	5
	Alcohol use disorder	5
	Cocaine dependence	4
Dermatology/autoimmune (n = 23)	Psoriasis	13
	Atopic dermatitis	6
	Giant cell arteritis	1
Rare genetic disorders (n = 10)	G6PD deficiency	6
	Wilson disease	2
	Crigler–Najjar syndrome	2
Hematology/coagulation (n = 13)	Sickle cell disease	5
	Stable coronary syndrome	1
	Aplastic anemia	1
Ophthalmology (n = 3)	Macular degeneration	3

Intermediate representation was observed in psychiatric and neurological disorders (112 entries, 6.4%) and cardiovascular conditions (106 entries, 6.1%), primarily focused on schizophrenia, major depressive disorder, coronary artery disease, atrial fibrillation, and hypertension.

Other categories were reported less frequently, including metabolic/endocrine (71 entries, 4.1%), musculoskeletal (44 entries, 2.5%), transplantation/immunology (35 entries, 2.0%), respiratory (32 entries, 1.8%), substance use (30 entries, 1.7%), dermatology/autoimmune (23 entries, 1.3%), hematology/coagulation (13 entries, 0.7%), rare genetic disorders (10 entries, 0.6%), and ophthalmology (3 entries, 0.2%).

### 3.2. Study phase and recruitment status

The distribution of study phases among the 1743 pharmacogenetic and pharmacogenomic clinical trials is shown in Table 2. Phase 1 studies were the most common, comprising 785 trials (45.0%), followed by Phase 2 with 456 trials (26.2%) and Phase 4 with 361 trials (20.7%). Phase 3 studies accounted for 222 trials (12.7%), while Early Phase 1 represented a smaller fraction of activity (25 trials, 1.4%). This indicates that the majority of pharmacogenetic research remains concentrated in early-phase exploratory investigations, although a substantial proportion has advanced into later phases.

**Table 2:** Distribution of pharmacogenetic and pharmacogenomic clinical trials by study phase (N = 1743).

Study phase	Number of trials (n)	Percentage (%)
Early phase 1	25	1.4
Phase 1	785	45.0
Phase 2	456	26.2
Phase 3	222	12.7
Phase 4	361	20.7

Recruitment status for the 1743 included trials is presented in Table 3. The majority were reported as completed (1201 trials, 68.8%), followed by trials with an unknown status (173 trials, 9.9%). A smaller proportion were classified as terminated (116 trials, 6.6%), recruiting (102 trials, 5.8%), or active but not recruiting (59 trials, 3.4%). Additional categories included not yet recruiting (42 trials, 2.4%), withdrawn (41 trials, 2.3%), suspended (5 trials, 0.3%), and enrolling by invitation (4 trials, 0.2%). Overall, the predominance of completed trials suggests that a large body of pharmacogenetic evidence is already available for analysis.

**Table 3:** Distribution of pharmacogenetic and pharmacogenomic clinical trials by recruitment status (N = 1743).

Status	Number of trials (n)	Percentage (%)
Completed	1201	68.8
Unknown	173	9.9
Terminated	116	6.6
Recruiting	102	5.8
Active, not recruiting	59	3.4
Not yet recruiting	42	2.4
Withdrawn	41	2.3
Suspended	5	0.3
Enrolling by invitation	4	0.2

### 3.3. Temporal trend in trial registration

**Figure 1.**
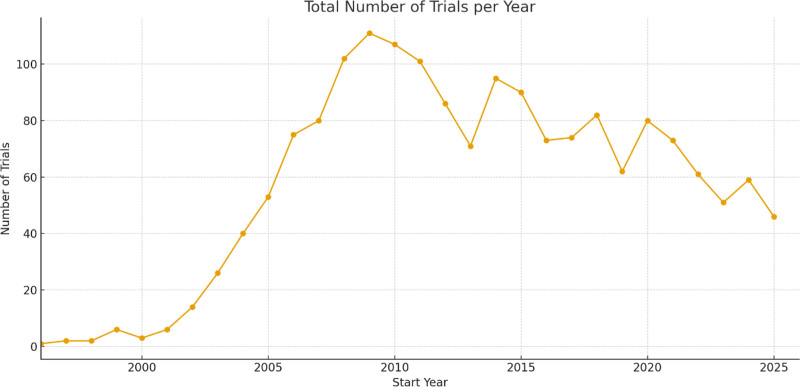
Temporal trend of pharmacogenetic and pharmacogenomic clinical trials by study phase (1996–2024). Line plot depicting the number of pharmacogenetic clinical trials registered each year among the 1743 studies included in the analysis.

The temporal trend of pharmacogenetic and pharmacogenomic clinical trial registrations is presented in Figure 1. Only isolated trials were registered before 2000, followed by a clear increase in activity during the early 2000s. Registrations peaked between 2008 and 2015, representing the most active period of pharmacogenetic trial initiation. Thereafter, the number of new registrations declined slightly but remained through 2024, indicating sustained though lower levels of research activity.

**Figure 2.**
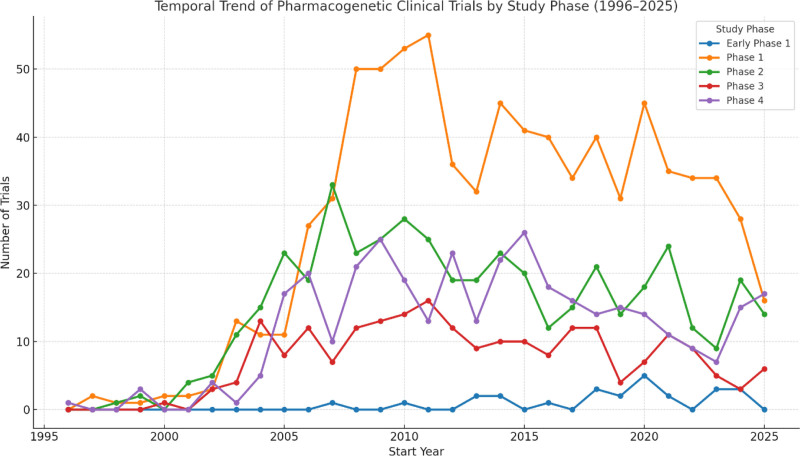
Temporal trend of pharmacogenetic clinical trials by study phase (1996–2025). Line plot showing the annual number of registered pharmacogenetic clinical trials by study phase. Phase 1 trials consistently outnumber later-stage studies, whereas phase 3 and phase 4 trials remain comparatively scarce.

The temporal distribution of pharmacogenetic clinical trials by study phase is presented in Figure 2. Phase 1 trials were consistently the most frequent, peaking during the early 2000s and remaining dominant throughout the observation period. Phase 2 trials demonstrated a moderate presence, while Phase 3 and Phase 4 trials were relatively uncommon, with only gradual increases over time. Early Phase 1 studies appeared sporadically and in small numbers. Collectively, these findings illustrate a strong emphasis on early-phase and mechanistic pharmacogenetic research, with fewer trials progressing to later stages of clinical evaluation.

### 3.4. Distribution of pharmacologic agents

The distribution of pharmacologic agents across the included trials is presented in Table 4. Oncology drugs were the most frequently represented, accounting for 430 study–drug pairs. Within this class, irinotecan was the predominant agent (n = 58), followed by capecitabine (n = 30) and oxaliplatin (n = 30), reflecting the central role of cytotoxic chemotherapy in genotype-guided oncology research. Antimicrobial agents were also common (n = 395), with itraconazole (n = 52), primaquine (n = 37), and rifampin (n = 22) among the most studied. These findings highlight the sustained importance of infectious disease pharmacogenomics, particularly in antifungal and antimalarial therapies.

**Table 4:** Distribution of main pharmacologic agents by drug class in pharmacogenetic clinical trials. Counts represent the number of study–drug pairs among 1611 pharmacologic interventions included. For each class, the top 3 agents are shown.

Main category (n)	Agent	Count
Oncology (n = 430)	Irinotecan	58
	Capecitabine	30
	Oxaliplatin	30
Antimicrobial (n = 395)	Itraconazole	52
	Primaquine	37
	Rifampin	22
CNS/analgesic (n = 242)	Midazolam	116
	Caffeine	35
	Dextromethorphan	21
Cardiovascular/hematology (n = 227)	Clopidogrel	38
	Digoxin	32
	Warfarin	32
Biologics/investigational (n = 209)	Bevacizumab	25
	Cetuximab	16
	BI-411034	8
Endocrine/metabolic (n = 43)	Metformin	23
	Rosiglitazone	9
	Pioglitazone	5
Immunosuppressant/transplant (n = 34)	Tacrolimus	20
	Azathioprine	9
	Cyclosporine	5

CNS = central nervous system.

Central nervous system and analgesic agents comprised 242 entries, dominated by midazolam (n = 116), caffeine (n = 35), and dextromethorphan (n = 21). The prominence of midazolam reflects its widespread use as a probe substrate in cytochrome P450 studies, underscoring its role in drug–gene interaction investigations. Cardiovascular and hematology-related drugs were represented in 227 entries, primarily clopidogrel (n = 38), digoxin (n = 32), and warfarin (n = 32), consistent with the emphasis on genotype-guided antithrombotic therapy and cardiac drug safety.

Biologics and investigational agents appeared in 209 entries, most frequently bevacizumab (n = 25), cetuximab (n = 16), and BI-411034 (n = 8). This category highlights the growing focus on targeted monoclonal antibodies and novel compounds in precision medicine. Endocrine and metabolic agents were less frequently studied (n = 43), with metformin (n = 23), rosiglitazone (n = 9), and pioglitazone (n = 5) leading this group, indicating an ongoing interest in pharmacogenomic determinants of diabetes and metabolic disease treatment. Immunosuppressants and transplant-related drugs were the least common (n = 34), with tacrolimus (n = 20), azathioprine (n = 9), and cyclosporine (n = 5) most often reported, reflecting their clinical relevance in transplant pharmacogenomics despite smaller trial numbers.

## 4. Discussion

This study examined the global landscape of pharmacogenetic clinical trials, characterizing their distribution by phase, therapeutic area, and drug class. Several patterns appeared evident. Oncology, psychiatry, and cardiovascular research seemed to dominate the evidence base, whereas metabolic and respiratory diseases, along with pediatric and geriatric conditions, appeared comparatively underrepresented. Methodological variability and persistent barriers to translation were also observed, suggesting both progress and continuing limitations in the field.

The analysis suggested that early-phase trials accounted for the majority of pharmacogenetic research, with Phase 1 and 2 together comprising around 70% of activity. In contrast, only 12.7% of studies were in Phase 3 and 20.7% in Phase 4. This imbalance may reflect a discipline that has built a strong mechanistic foundation but has yet to demonstrate comparable growth in confirmatory research. Several factors could help explain this gap. Surveys have suggested that many healthcare providers feel unprepared to interpret pharmacogenetic results,^[20,21]^ which may reduce clinical demand for large-scale validation. Financial considerations also appear important. Although multicenter investigations such as the PREPARE trial have shown reductions in adverse drug reactions, uncertainties regarding cost-effectiveness and inconsistent reimbursement models continue to constrain adoption.^[12,22]^ Variation in the uptake of guidelines from groups such as Clinical Pharmacogenetics Implementation Consortium may further contribute to uneven progress.^[23,24]^ Collectively, these issues suggest why exploratory studies have expanded while confirmatory late-phase trials remain limited.

Furthermore, the trial activity-over time did not appear to rise steadily but instead showed signs of modest decline in recent years. This trend may indicate that pharmacogenetic research is sensitive to systemic influences such as funding cycles, regulatory priorities, or shifting perceptions of utility. Although the precise causes remain uncertain, the absence of continuous growth seems to raise questions about the sustainability of investment in the field.

Therapeutic distribution also revealed imbalances. Oncology accounted for a disproportionately large share of pharmacogenetic trials. This concentration likely reflects the nature of oncology drug development, where high costs and risks create strong incentives to identify predictive biomarkers and optimize patient selection. Regulatory frameworks may have reinforced this emphasis, as more than 140 U.S. Food and Drug Administration -approved drugs currently include pharmacogenomic information in their labeling.^[25,26]^ The convergence of biological plausibility, regulatory attention, and commercial investment therefore appears to have positioned oncology as the principal setting for pharmacogenetic application.^[27,28]^

In contrast, conditions such as diabetes, asthma, and chronic obstructive pulmonary disease appeared underrepresented. This pattern may reflect the limited number of validated pharmacogenetic biomarkers in these areas, as well as challenges in recruiting representative cohorts and reduced commercial incentives for therapies already widely available.^[29,30]^ Gene–drug associations influencing corticosteroid or β2-agonist responsiveness in asthma have been described,^[31,32]^ yet translation into large-scale trials has been limited. Furthermore, the restricted ancestral diversity of existing evidence, with most studies conducted in populations of European descent, may reduce the generalizability of findings across global populations.^[33,34]^ Expanding research into underrepresented conditions and more diverse populations is likely to be essential if pharmacogenetics is to achieve broader clinical relevance.

Trial activity also appeared concentrated in a limited set of pharmacologic agents and pharmacogenes. Oncology drugs provided the clearest examples, with UGT1A1 variation predicting irinotecan toxicity and dihydropyrimidine dehydrogenase (DPYD) deficiency influencing fluoropyrimidine intolerance. These associations have been validated across several cohorts, incorporated into guidelines, and, in some contexts, prompted adoption of preemptive testing.^[35,36]^ Cardiovascular agents such as clopidogrel and warfarin remain important reference cases of successful translation, with pharmacogenetic dosing strategies supported by randomized trials.^[20,37]^ Antimicrobials such as primaquine, which requires CYP2D6 activation, and psychiatric agents metabolized by CYP2D6 and CYP2C19 provide further clinically actionable examples.^[23,38]^ Overall, most evidence has focused on a narrow group of enzymes, particularly CYP2D6, CYP2C19, CYP3A4/5, CYP2C9, UGT1A1, and DPYD. While these pathways have yielded some of the most consistent and clinically relevant associations, many other substrates remain underexplored.

Despite such successes, methodological limitations appear to persist. Many pharmacogenetic trials rely on pharmacokinetic endpoints, such as drug metabolism rates or plasma concentrations, which may not always translate into patient-level outcomes. The case of warfarin illustrates this challenge: genotype-guided dosing improved international normalized ratio control in some studies, yet large trials such as COAG did not demonstrate significant reductions in thromboembolic or bleeding events.^[39,40]^ Recruitment and statistical power also continue to pose difficulties, as pharmacogenetic effects often require large and ancestrally diverse cohorts to detect. Rare variants and uneven allele frequencies across populations further complicate generalizability.^[19,41]^ In addition, the rapid pace of discovery may sometimes lead to premature incorporation of emerging variants into dosing algorithms before adequate validation.^[42]^

Translation into practice seems further constrained by systemic barriers. Incorporating pharmacogenetic testing into routine workflows requires adaptation of electronic health records and decision support tools. Evidence suggests that such tools can improve adherence to guideline-based prescribing,^[43,44]^ although implementation remains inconsistent due to differences in system design and interoperability.^[45,46]^ The knowledge and attitudes of healthcare professionals also appear central. Many clinicians report limited preparedness to apply pharmacogenetic information, underscoring the need for structured education across the healthcare workforce.^[47,48]^ Pharmacists and other front-line providers may play an important intermediary role in this process.^[49,50]^

Finally, economic considerations remain a significant constraint. While pharmacogenetic testing has the potential to reduce adverse drug reactions and improve adherence, evidence for cost-effectiveness remains variable, and reimbursement policies are inconsistent across settings.^[51,52]^ Without sustainable financial models, widespread adoption may remain difficult to achieve.

In summary, pharmacogenetic clinical trials appear to remain concentrated in early phases, a limited range of therapeutic areas, and a small group of pharmacogenes. Oncology seems to have benefited from the strongest evidence and regulatory attention, whereas other fields appear comparatively less represented. Methodological and translational challenges, including reliance on intermediate endpoints, limited ancestral diversity, and uneven integration into healthcare systems, continue to constrain the broader impact of the field. Future progress is likely to depend on extending research into underrepresented conditions and populations, refining trial designs, and strengthening infrastructure, education, and economic evaluation. These measures may be necessary for pharmacogenetics to move from specialist programs toward integration into routine clinical practice.

## Author contributions

**Conceptualization:** Mohammed Aldurdunji.

**Data curation:** Ahmed Ashour, Fahad Alshehri, Nasser Alorfi.

**Formal analysis:** Ahmed Ashour.

**Investigation:** Mohammed Aldurdunji, Ahmed Ashour, Fahad Alshehri, Nasser Alorfi.

**Methodology:** Fahad Alshehri.

**Project administration:** Mohammed Aldurdunji, Fahad Alshehri.

**Supervision:** Mohammed Aldurdunji.

**Validation:** Ahmed Ashour, Fahad Alshehri, Nasser Alorfi.

**Visualization:** Ahmed Ashour, Fahad Alshehri, Nasser Alorfi.

**Writing – original draft:** Mohammed Aldurdunji, Ahmed Ashour, Fahad Alshehri, Nasser Alorfi.

**Writing – review & editing:** Nasser Alorfi.
